# Understanding genomic diversity, pan-genome, and evolution of SARS-CoV-2

**DOI:** 10.7717/peerj.9576

**Published:** 2020-07-17

**Authors:** Arohi Parlikar, Kishan Kalia, Shruti Sinha, Sucheta Patnaik, Neeraj Sharma, Sai Gayatri Vemuri, Gaurav Sharma

**Affiliations:** Institute of Bioinformatics and Applied Biotechnology (IBAB), Bengaluru, Karnataka, India

**Keywords:** COVID-19, SARS, Coronavirus, Pandemic, Viral disease, Genome, Bioinformatics, Genomics, Mutations

## Abstract

Coronovirus disease 2019 (COVID-19) infection, which originated from Wuhan, China, has seized the whole world in its grasp and created a huge pandemic situation before humanity. Since December 2019, genomes of numerous isolates have been sequenced and analyzed for testing confirmation, epidemiology, and evolutionary studies. In the first half of this article, we provide a detailed review of the history and origin of COVID-19, followed by the taxonomy, nomenclature and genome organization of its causative agent Severe Acute Respiratory Syndrome-related Coronavirus-2 (SARS-CoV-2). In the latter half, we analyze subgenus *Sarbecovirus* (167 SARS-CoV-2, 312 SARS-CoV, and 5 Pangolin CoV) genomes to understand their diversity, origin, and evolution, along with pan-genome analysis of genus *Betacoronavirus* members. Whole-genome sequence-based phylogeny of subgenus *Sarbecovirus* genomes reasserted the fact that SARS-CoV-2 strains evolved from their common ancestors putatively residing in bat or pangolin hosts. We predicted a few country-specific patterns of relatedness and identified mutational hotspots with high, medium and low probability based on genome alignment of 167 SARS-CoV-2 strains. A total of 100-nucleotide segment-based homology studies revealed that the majority of the SARS-CoV-2 genome segments are close to Bat CoV, followed by some to Pangolin CoV, and some are unique ones. Open pan-genome of genus *Betacoronavirus* members indicates the diversity contributed by the novel viruses emerging in this group. Overall, the exploration of the diversity of these isolates, mutational hotspots and pan-genome will shed light on the evolution and pathogenicity of SARS-CoV-2 and help in developing putative methods of diagnosis and treatment.

## Introduction

The emergence of coronavirus disease 2019 (COVID-19) has sent people from across the world into a state of high alert, and they are trying to survive complete or partial lockdowns (Lescure et al., 2020). The causative agent of this pandemic is a novel Severe Acute Respiratory Syndrome (SARS) Coronavirus, Severe Acute Respiratory Syndrome-related Coronavirus-2 (SARS-CoV-2). Since 2000, the world has witnessed two major coronavirus outbreaks: the first, of SARS, caused by SARS-CoV, in 2002 in China (Zhong et al., 2003), and the second, of Middle East Respiratory Syndrome (MERS) in 2012 in Saudi Arabia (Zaki et al., 2012). Amongst coronaviruses, SARS-CoV and MERS are known as highly pathogenic viruses that cause pneumonia and respiratory system infections (Coleman & Frieman, 2014). Besides these, several other low pathogenic strains are also known that cause mild respiratory diseases and infect majorly the upper respiratory tract (Han et al., 2020). After the diagnosis of the first reported case in Wuhan, China, and genome sequencing of the Wuhan Hu-1 strain (SARS-CoV-2 reference genome), scientists across the world are striving to develop vaccines and diagnostic kits and understand its evolution and epidemiology. Several potential drug molecules to combat COVID-19 are undergoing clinical trials. As no vaccine or drugs are available at present, the only ways known to contain transmission are social distancing, regular washing of hands, and covering mouth and nose while coughing or sneezing, thereby, preventing direct or indirect physical contact and containing the transfer of infected respiratory droplets.

This study comprises a detailed overview of the history and origin of COVID-19 along with the taxonomy, nomenclature and genome structure of its causative agent SARS-CoV-2. Following that, we analyze 167 SARS-CoV-2, 312 SARS-CoV and five Pangolin CoV genomes to study their genomic variability, evolution and mutation hotspots. We also characterize the pan-genome of the genus *Betacoronavirus* (including the recently sequenced Pangolin CoV) to classify their proteins-coding regions into core, accessory, and unique categories within each genome group.

## Taxonomy and Nomenclature

Coronaviruses are members of family *Coronaviridae* that include enveloped positive sense ssRNA containing viruses, taxonomically classified amongst order *Nidovirales* in the realm *Riboviria*. Like other viruses, they thrive in the gray area of living and non-living as they do not have the machinery to survive outside a living host cell. Therefore, they cannot replicate outside the host. The Riboviruses or realm *Riboviria* members replicate by utilizing RNA-dependent RNA-polymerases (RdRps). Their genetic material that is, RNA serves as messenger RNA (mRNA), directly translating into proteins and undergoes genetic recombination in the presence of another viral genome in the host cell (Barr & Fearns, 2010).

Members of order *Nidovirales* have a positive sense linear (capped and polyadenylated) RNA molecule, known for causing severe infections. The word “Nido” stands for “nest” implying that all Nidoviruses express subgenomic mRNAs (sgRNA) in a nested form. They are known to infect hosts within three important classes in Vertebrates, namely mammals (*Mammalia*), birds (*Aves*) and fishes (*Pisces*). Coronaviruses (family *Coronaviridae*; subfamily *Orthocoronavirinae*) commonly infect both mammals and birds; Torovirus (family *Tobaniviridae*; subfamily *Torovirinae*) infect specifically mammals and Bafinivirus (family *Tobaniviridae*; subfamily *Piscanivirinae*) infect fishes. Coronavirus virions are spherical, Torovirus are crescent-shaped, and Bafinivirus are rod-like; however, all members of this family are adorned with a crown or club-shaped surface proteins called the Spike proteins. Because of their crown-like morphology observed in electron micrographs, they are named Coronaviruses. Family *Coronaviridae* has a single subfamily *Orthocoronavirinae* and its members form a monophyletic clade. They are further classified into four defined genera based on phylogenetic studies of conserved genome regions and their serological cross-reactivity: *Alphacoronavirus, Betacoronavirus, Gammacoronavirus* and *Deltacoronavirus*. Under genus *Betacoronavirus*, five lineages diverge: Lineage A (subgenus: *Embecovirus*) includes HCoV-OC43 and HCoV-HKU1, Lineage B (subgenus: *Sarbecovirus*) includes SARS-CoV and SARS-CoV-2, Lineage C (subgenus: *Merbecovirus*) contains Bat coronavirus BtCoV-HKU4 and BtCoV-HKU5, Lineage D (subgenus: *Nobecovirus*) includes Rousettus Bat coronavirus BtCoV-HKU9 and lineage E (subgenus: *Hibecovirus)* includes Bat Hp-betacoronavirus/Zhejiang2013. The subgenus *Sarbecovirus* members tend to undergo deep recombination events leading to the formation of new alleles (Boni et al., 2020) and zoonotic transfer.

## Origin and History of the Coronavirus Infections

The first Coronavirus associated disease was described in 1931 (Peiris, 2012). The viruses were first isolated from humans in the UK and USA around the same time. The first isolated specimen was B814, taken from a boy exhibiting symptoms of the common cold in 1960 and cultured in human embryonic tracheal organ culture (Tyrrell & Bynoe, 1966). The specimen was deemed distinct from Adeno-, Entero- or Rhinoviruses, being ether labile and propagated only in organ cultures (Kendall, Bynoe & Tyrrell, 1962; Tyrrell & Bynoe, 1966). Later, the gradual discoveries of 229E in 1966 (Hamre & Procknow, 1966), OC43 in 1967 (McIntosh, Becker & Chanock, 1967), SARS-CoV in 2002, Bovine CoV in 2006, MERS-CoV in 2012 and SARS-CoV-2 in 2019 (Zhu et al., 2020) were major significant steps in coronavirus history.

Coronaviruses have been associated with mild respiratory infections and cold in humans and animals (specifically poultry and livestock). These viruses were not considered treacherous until the SARS epidemic that emerged in China in November 2002. The associated SARS-CoV species were first classified as a separate clade under *Betacoronavirus* after this outbreak, post which new species including those of human coronaviruses (hCoVs) have been identified. The 2002 SARS epidemic ultimately infected 8,096 people, with 774 deaths. Later, MERS-CoV emerged in Saudi Arabia in September 2012, causing 2,494 confirmed cases of infection and 858 deaths across 27 countries (https://www.who.int/emergencies/diseases/en/).

The current pandemic, COVID-19, is caused by the virus, initially designated “2019 novel coronavirus” or “2019-nCoV”, later re-named SARS-CoV-2, segregating it as a novel SARS species (Huang et al., 2020). Now, it forms a new clade in the subgenus *Sarbecovirus* and is established as the 7th member of the family which infects humans (Simmonds et al., 2017; Gorbalenya et al., 2020; Zhu et al., 2020). The first patients of COVID-19 were identified in late December 2019 when several local health centers reported clusters of patients with pneumonia caused by an unknown pathogen, epidemiologically linked to living animal and seafood wholesale market in Wuhan. The Chinese Center for Disease Control and Prevention called a rapid action team for the epidemiological investigation. Bronchoalveolar-lavage fluids were propagated on human respiratory tract epithelial cell cultures for 4–6 weeks. Extracted nucleic acid was identified as viral RNA by real-time reverse transcription PCR targeting the RdRp region of pan-BetaCoV. The electron micrographs revealed the presence of distinctive spikes of length 9–12 nm, establishing morphological resemblance with the *Coronaviridae* family (Zhu et al., 2020). Following this, SARS-CoV-2 infection has been spreading worldwide. As of 29 April 2020 (submission date), 213 countries and territories around the world are under surveillance, with more than 3.15 million confirmed cases and over 218,490 reported fatalities, and these numbers continue to exponentially increase day by day.

Since December 2019, numerous SARS-CoV-2 genomes have been sequenced, which allows researchers to address many critical questions regarding its origin, transmission, epidemiology, and most importantly to design vaccines and viral detection kits. Viral genome sequencing and multiple sequence alignment of SARS-CoV-2 genomes have revealed its identity with Bat CoV RaTG13 (MN996532) and Pangolin-CoV (Cui, Li & Shi, 2019; Boni et al., 2020; Rehman et al., 2020). A comparative phylogenetic study revealed that the Pangolin-CoV genes shared a high level of sequence identity with SARS-CoV-2 genes, specifically orf1b, the spike (S), orf7a and orf10 genes (Zhang, Wu & Zhang, 2020). Higher identity of S protein sequence implies the functional similarity between Pangolin-CoV and SARS-CoV-2 as compared to the RaTg1 strain (Zhang, Wu & Zhang, 2020) further suggesting Pangolin as an intermediate host for infection and natural reservoir of SARS-CoV-2-like strains.

## Coronavirus genome Organization

Coronaviruses belong to family *Coronaviridae* and comprise enveloped viruses that replicate in the cytoplasm of animal host cells; distinguished by the presence of a single-stranded positive-sense RNA genome (about 30 kb in length) (Marra et al., 2003). Coronaviruses possess the largest genomes among all known RNA viruses (Fehr & Perlman, 2015), ranging from 25.32 kb in Porcine Deltacoronavirus PD-CoV to 31.775 kb in Beluga whale coronavirus SW1 (information available on NCBI Virus webpage https://www.ncbi.nlm.nih.gov/labs/virus/). SARS-CoV-2 is a spherical or pleomorphic enveloped particle, containing single-stranded, positive-sense RNA associated with a nucleoprotein, lying inside a capsid composed of matrix protein (Lai et al., 2020).

The SARS-CoV-2 reference genome (NC_045512; Wuhan Hu-1 strain) is 29,903 nucleotides long, which consists of A (8,954 nt, 29.94%), G (5,863 nt, 19.61%), C (5,492 nt, 18.37%), T (9,594 nt, 32.08%). Compared to SARS-CoV-2, the SARS-CoV reference genome (NC_004718; Tor1 strain) is a little larger, that is, 29,751 nucleotides and its nucleotide composition is marginally different: A (8,481 nt, 28.50), G (6,187 nt, 20.80%), C (5,940 nt, 19.97%), T (9,143 nt, 30.73%). We identified that the GC content (average of all genomes under this study) of SARS-CoV, SARS-CoV-2, and Pangolin CoV is 40.81%, 38% and 38.52% respectively. Like other *Betacoronavirus* members, this genome contains two flanking untranslated regions (UTRs), 5′-UTR (265 nt) and 3′-UTR (229 nt) sequences.

A typical CoV genome contains at least six ORFs (Graham et al., 2008). The genomes of all coronaviruses usually encode four well-conserved and characterized structural proteins: S (spike), E (envelope), M (membrane) and N (nucleocapsid) (Graham et al., 2008; Fuk-Woo Chan et al., 2020), encoded by sgRNA 2, 4, 5 and 9a respectively present at 3′end and sharing 1/3 of the genome in SARS-CoV (Graham et al., 2008).

*SARS-CoV-2 Proteins:* The SARS-CoV-2 genome has 12 protein-coding regions, which encode two categories of proteins: first, Structural proteins, which give characteristic structure to the virus and are involved in viral entry, and second, Non-structural (NS) or accessory proteins, which help in viral replication (Marra et al., 2003; Graham et al., 2008; Hu et al., 2018; Fuk-Woo Chan et al., 2020). Most of the information available so far about these proteins is based on SARS-CoV and other members of the genus *Betacoronavirus*.

## Structural Proteins

### Spike protein

The S protein is a 1,273 aa trimeric, cell-surface glycoprotein consisting of two subunits (S1 and S2), encoded by the gene S. The S1 subunit is responsible for receptor binding (Hu et al., 2018). This is also important for mediating the fusion of viral and host membranes. Both these processes are critical for virus entry into host cells (Tan, Lim & Hong, 2005). SARS-CoV-2 has a polybasic cleavage site RRAR, at the junction of S1 and S2 subunits, which enables effective cleavage by Furin and other proteases (Andersen et al., 2020; Zhang, Wu & Zhang, 2020). Furthermore, one proline residue is also inserted at the leading cleavage site of SARS-CoV-2, making “PRRA”, the final inserted sequence. Comparison of this site within different *Betacoronavirus* members revealed that the insertion of a Furin cleavage site at the S1–S2 junction of SARS-CoV-2 enhances cell-cell fusion (Follis, York & Nunberg, 2006; De Haan et al., 2008; Andersen et al., 2020). Similarly, an effective cleavage of the polybasic cleavage site in Hemagglutinin esterase protein facilitated the inter-species transmission of MERS-like coronaviruses from bats to humans (Andersen et al., 2020). Majorly, variations in the S protein are responsible for two attributes, tissue tropism and host ranges of different CoVs (Hu et al., 2018). It was observed that S protein underwent several drastic changes during the viral infection. The S protein of SARS-CoV-2 is more vulnerable to mutations, especially in the amino acids associated with the spike protein-cell receptor interface. Interestingly, the amino acid sequence represented ~19% changes with four major insertions and ~81% sequence similarity in contrast to SARS-CoV (Ahmed, Quadeer & McKay, 2020; Rehman et al., 2020).

### Nucleocapsid protein

N proteins are 419 aa long phosphoproteins weighing ~46kDa. These have helix binding properties. Coronavirus N proteins possess three easily distinguishable and highly conserved domains; the N-terminal domain (NTD) (N1b), the C-terminal domain (CTD) (N2b) and the N3 region (Grossoehme et al., 2009; Cong et al., 2019). The N protein plays a vital role in virion structure formation as it is localized in both the replication-transcription region and the ERGIC (Endoplasmic reticulum-Golgi apparatus Intermediate Compartment), the site of virion assembly (Tok & Tatar, 2017). The N protein of SARS-CoV is primarily expressed during the early stages of SARS-CoV infection (Surjit & Lal, 2008; Grossoehme et al., 2009; Cong et al., 2019). It is important as a diagnostic marker as it induces a strong immune response (Hu et al., 2018). Evolutionary analysis has shown 89–91% sequence homology between the N proteins in the SARS-CoV and Bat SL-CoV (Hu et al., 2018; Ahmed, Quadeer & McKay, 2020). The N protein can bind to Nsp3 protein to help bind the genome to replication/transcription complex (RTC) (Cong et al., 2019) and package the encapsulated genome into virions (Chen, Liu & Guo, 2020). IBV, SARS CoV and MHV N protein undergo phosphorylation and allow discrimination of non-viral mRNA binding from viral mRNA binding (Cong et al., 2019). N protein is also an antagonist of interferon (IFN) (Kopecky-Bromberg et al., 2007) and virus-encoded repressor of RNA interference (RNAi), therefore, it appears to benefit the viral replication (Chen, Liu & Guo, 2020).

### Membrane protein

The most abundant structural protein in the genome is the membrane (M) glycoprotein, which spans across the membrane bilayer three times. Thus, M glycoproteins have three transmembrane regions, leaving a short NH2-terminal domain outside the virus and a long COOH terminus (cytoplasmic domain) inside the virion (Tok & Tatar, 2017; Mousavizadeh & Ghasemi, 2020). The M protein plays a key role in regenerating virions in the cell. M proteins undergo glycosylation in the Golgi apparatus which is crucial for the virion to fuze into the cell and to make antigenic proteins. As mentioned before, N protein forms a complex by binding to genomic RNA and M protein triggers the formation of interacting virions in the endoplasm (Tok & Tatar, 2017).

### Envelope glycoprotein

E glycoproteins are composed of approximately 76–109 amino acids in other coronavirus species. E protein plays a crucial role in the assembly and morphogenesis of virions within the host. About 30 amino acids in the N-terminus of the E protein enable binding to the viral membranes. Co-expression of E and M proteins with mammalian expression vectors enable the formation of virus-like structures within the cell (Tok & Tatar, 2017).

## Non-structural (accessory) Proteins

### ORF1ab and ORF1a

The SARS 5′ proximal gene 1 (~22 kb) comprises two long overlapping open reading frames, orf1a and orf1ab, encoding for polyproteins 1a and 1ab. These polyproteins are cleaved by viral proteases that is, PL^pro^ (papain-like protease) and 3CL^pro^ (chymotrypsin-like protease) into 16 non-structural proteins, Nsp1–Nsp16. Expression of orf1ab involves a (−1) ribosomal frameshift upstream of orf1a stop codon, thus forming the full-length ORF1ab. ORF1a polyprotein of ~500 kDa encodes for Nsp 1–11, while ORF1ab polyprotein of ~800 kDa encodes for all Nsp1–16. The ORF1a and ORF1ab polyproteins undergo post-translational modifications to form mature proteins and hence are not detected during infection (Graham et al., 2008; Lei, Kusov & Hilgenfeld, 2018). A brief description of these proteins is provided below:

- *Nsp1:* SARS-CoV Nsp1 is an N-terminal protein coded by the first gene of ORF1ab, which plays an important role in SARS-CoV pathogenesis by inhibiting the host gene expression via binding to the small subunit of the ribosome and then truncating the translation activity. Nsp1 induces endonucleolytic RNA cleavage of a capped host mRNA, making it translationally incompetent (Tanaka et al., 2012). Nsp1 inhibits the type-1 IFN expression and other antiviral signaling pathways, thus suppressing the innate immune system. The viral mRNA is resistant to Nsp1-mediated RNA cleavage in the infected host cells, however, the mechanism through which it achieves this is unknown (Tanaka et al., 2012).
- *Nsp2:* the function of Nsp2 in SARS-CoV is unknown. The experimental results show that the deletion of the *nsp2* gene from MHV and SARS-CoV was tolerated with modest growth and some RNA defect. Also, there was no observable effect on the polyprotein processing in the mutants. Another evidence shows that neither the Nsp2 protein nor the *nsp2* gene is involved in pathogenesis (Graham et al., 2005).
- *Nsp3:* the multi-domain SARS-CoV Nsp3 is a 215 kDa glycosylated transmembrane multidomain protein involved in viral replication and transcription. It may serve as a scaffolding protein for numerous other proteins (Angelini et al., 2013). The organization of various Nsp3 domains differs amongst the Coronavirus genomes. However, eight domains are common to all CoVs: ubiquitin-like domain 1 (Ubl1), the glutamate-rich acidic domain also known as “hypervariable region”, an X macrodomain, ubiquitin-like domain 2 (Ubl2), PL2^pro^ (papain-like protease 2), ectodomain 3Ecto, also known as “zinc-finger domain”, and domains Y1 and CoV-Y (unknown functions). Nsp3 releases itself, Nsp1 and Nsp2 from the polyproteins. It alters the post-translational modification of host proteins to antagonize the innate immune response by de-MARylating, de-PARylating, de-ubiquitinating, or de-ISGylating the host proteins. Meanwhile, Nsp3 modifies itself by the N-glycosylation of the ectodomain and can also interact with host proteins (such as RCHY1) to enhance viral survival (Lei, Kusov & Hilgenfeld, 2018).
- *Nsp4:* it is known that Coronaviruses induce double-membrane vesicles (DMVs). Any alteration in the DMVs’ morphology impairs the RNA synthesis and therefore growth of Nsp4 mutants (Gadlage et al., 2010).
- *Nsp5:* the Nsp5 protease also known as 3CL^pro^ or M^pro^, cleaves the Nsp peptides at 11 cleavage sites (Stobart et al., 2013). Nsp5 of Porcine Deltacoronavirus (PDCoV) is a type I IFN antagonist. It disrupts the IFN signaling pathway by cleaving the NF-κB essential modulator (NEMO), thus impairing the host’s ability to activate the IFN response (Zhu et al., 2017).
- *Nsp6:* Nsp6, along with Nsp3 and Nsp4, has membrane proliferation ability. Thus, it can induce perinuclear vesicles localized around the microtubule-organizing center and DMV formation (Angelini et al., 2013). The coronavirus Nsp6 protein restricts the autophagosome expansion either directly or indirectly through starvation or chemical inhibition of MTOR signaling (Cottam, Whelband & Wileman, 2014).
- *Nsp7:* the Nsp12 RNA-dependent RNA polymerase cannot act on its own and depends upon stimulation by a complex of Nsp7 and Nsp8. Thus, Nsp7 acts as one of the cofactors along with Nsp8 to stimulate Nsp12. It is hypothesized that Nsp7 and Nsp8 heterodimers stabilize the Nsp12 regions involved in RNA binding; also, Nsp8 acts as a minor RdRp (Kirchdoerfer & Ward, 2019).
- *Nsp8:* Nsp8 is a 22 kDa non-canonical polymerase shown to be capable of de novo RNA synthesis with low fidelity on single-stranded RNA templates. The ‘main’ RdRp in Coronaviruses is the Nsp12 which employs a primer-dependent initiation mechanism. These observations led to a hypothesis that Nsp8 would act as an RNA primase and synthesize a short primer that will be extended by Nsp12 (Te Velthuis, Van den Worm & Snijder, 2012).
- *Nsp9:* Nsp9 is an RNA-binding subunit in the replication complex in all coronaviruses (Zeng et al., 2018).
- *Nsp10:* SARS-CoV Nsp10 binds to and stimulates the exoribonucleases, Nsp14, and Nsp16, thus playing an important role in the replication-transcription complex (RTC) formation (Bouvet et al., 2014). Nsp10 acts as an essential co-factor in triggering Nsp16 2′*O*-MTase activity, which suggests its involvement in the regulation of capping of viral RNA (Lugari et al., 2010).
- *Nsp11:* the exact function of Nsp11 in *Coronaviridae* is unknown. Arterivirus Nsp11 was identified as NendoU (Nidoviral uridylate-specific endoribonucleases) that plays a role in the viral life cycle. Nsp11 could be essential to produce helicase (Woo et al., 2005).
- *Nsp12:* Nsp12 is a 102 kDa RNA-dependent RNA polymerase (RdRp) featuring all conserved motifs of the known RdRps. It is the most conserved protein in coronaviruses and assumes a center stage in the viral RTC. The Nsp7/Nsp8 complex enhances the binding of Nsp12 to RNA. Nsp8 is the second, non-canonical RdRp in coronaviruses (Subissi et al., 2014).
- *Nsp13:* Nsp13 is a 66.5 kDa multi-functional protein. The N terminal has a zinc-binding domain while the C-terminal harbors a helicase domain-containing conserved motif of superfamily-1 helicases (Subissi et al., 2014).
- *Nsp14:* Nsp14 is a 60 kDa bifunctional enzyme. Its N-terminal is a 3′–5′ exoribonuclease involved in the mismatch repair system, improving the fidelity of virus replication via RNA proofreading. Yeast trans-complementation studies have shown that the C-terminal domain of SARS-CoV Nsp14 is an S-adenosylmethionine-dependent guanine-N7-methyltransferase (MTase) (Subissi et al., 2014; Zeng et al., 2016) with no RNA sequence specificity (Subissi et al., 2014).
- *Nsp15:* SARS-CoV Nsp15 is a uridine specific ribonuclease that cleaves the 3′ of uridylates, producing 2′–3′ cyclic phosphate ends (Subissi et al., 2014).
- *Nsp16:* Nsp16 is a 2′-*O*-Methyl Transferase (Zeng et al., 2016) and requires Nsp10 as a stimulatory factor to exhibit its MTase activity (Chen et al., 2011). Eukaryotic mRNA has a unique 5′ cap; to evade the host machinery, the viral RNA must be made indistinguishable from the host mRNA by capping the viral RNA (Menachery, Debbink & Baric, 2014). Both Nsp14 and Nsp16 are involved in the modification of viral RNA cap structure (Zeng et al., 2016) to maintain the viral RNA stability, ensure protein translation and immune escape (Lugari et al., 2010; Subissi et al., 2014). The SARS-CoV genome encodes two SAM-dependent methyltransferases (Zeng et al., 2016); Nsp14 N7 methyltransferase, and Nsp16 2′-O-methyl transferase, that methylates the RNA at N7 of guanosine and ribose 2′O sites, respectively (Chen et al., 2011). This process also involves Nsp10 for stabilizing the SAM binding region and RNA binding of Nsp16 (Subissi et al., 2014).

### ORF3a protein

The orf3a gene lies between S and E genes and encodes for a transmembrane protein (McBride & Fielding, 2012). SARS-CoV-2 ORF3a protein shows 97.82% homology to NS3 of Bat coronavirus RaTG13 and 72% homology to SARS-CoV ORF3a protein (Issa et al., 2020). It is localized to the cell membrane and partly to the ER and the Golgi perinuclear space in the host cell. ORF3a is known to activate PERK (PKR-like ER kinase) signaling pathway through which the viral particles escape ER-associated degradation and the resulting ER stress also induces apoptosis. ORF3a of both SARS-CoV and SARS-CoV-2 have an APA3_viroporin (a pro-apoptosis protein) conserved domain (McBride & Fielding, 2012; Issa et al., 2020). ORF3a has been shown to activate NF-κB and JNK. This leads to the upregulation of the chemokine named RANTES (Regulated upon Activation, Normal T Cell Expressed and Secreted) along with IL-8 in A549 and HEK293T cells (Liu et al., 2014). The extracellular N-terminus of protein can evoke a humoral immune response. Binding of ORF3a protein to caveolin-1 may be required for viral uptake and release (Liu et al., 2014). ORF3a can augment the activation of the p38 MAPK pathway and induce the mitochondria to leak inducing apoptosis (Liu et al., 2014).

### ORF6 protein

SARS-CoV ORF6/Protein 6 is a 63-aa polypeptide. It has an amphipathic 1–40 aa N-terminal portion and a highly polar C-terminal. The amino acid residues 2–32 in the N-terminal form an α-helix which is embedded in the cell membrane. There are two signal sequences in the C-terminal; aa 49–52 sequence YSEL targets proteins for incorporating into endosomes and the acidic tail which signals ER export. This protein is localized in the ER and Golgi apparatus. ORF6 is incorporated into VLPs, when co-expressed with SARS-CoV S, M and E structural proteins. It enhances viral replication, thus serving an important role in pathogenesis during SARS-CoV infection (Liu et al., 2014). ORF6 is also well known as a β-interferon antagonist; its overexpression inhibits nuclear import of STAT1 in IFN-β treated cells. Along with ORF3b and N protein, the SARS-CoV ORF6 inhibits activation of IRF-3 via phosphorylation and binding of IRF-3 to a promoter with IRF-3 binding sites. IRF-3 is an important protein for the expression of IFN. ORF6 may disturb the ER/Golgi transport necessary for the interferon response (Kopecky-Bromberg et al., 2007). During SARS-CoV infection, the C-terminal domain of ORF6 modulates the host protein nuclear transport and type-I interferon signaling, thus playing an important role in immune evasion (Liu et al., 2014).

### ORF7a protein

SARS-CoV ORF7a/ Protein 7a is a 122 aa type-I transmembrane protein (Liu et al., 2014). It consists of 15 aa N terminal signal peptide sequence, an 81 aa ectodomain, 21 aa C terminal transmembrane domain, and a cytoplasmic tail of 5 aa residues. The ectodomain of ORF7a binds to human LFA-1 (lymphocyte function-related antigen 1) on Jurkat cells via the alpha integrin-I domain of LFA-1. This suggests that probably LFA-1 is a binding factor or receptor for SARS-CoV on human leukocytes (McBride & Fielding, 2012). SARS-CoV ORF7a is localized in the ER-Golgi Intermediate Compartment (ERGIC), the assembly point of coronaviruses. It interacts with M and E structural proteins, indicating a possible role in viral assembly during SARS-CoV replication. In mammalian cells infected with SARS-CoV, ORF7a has been known to induce caspase-dependent apoptosis by cleaving PARP (poly(ADP-ribose) polymerase), an apoptotic marker. Its pro-apoptotic nature is a result of the interaction between its transmembrane domain with Bcl-X_L_, an anti-apoptotic protein of the Bcl-2 family (Liu et al., 2014). Like ORF3a, overexpression of ORF7a activates NF-κB and c-Jun N-terminal kinase (JNK), augmenting the production of pro-inflammatory cytokines such as interleukin 8 (IL-8) and RANTES (Liu et al., 2014). ORF7a overexpression induced apoptosis can occur via a caspase-3 dependent pathway as well as p38 MAPK pathway (McBride & Fielding, 2012).

### ORF7b protein

SARS-CoV ORF7b/Protein 7b is a 44 aa long, highly hydrophobic, integral transmembrane protein with a luminal N-terminal and cytoplasmic C-terminal. The expression of ORF7a is reduced significantly when the orf7a start codon (upstream of 7b) is mutated to a strong Kozak sequence or when an additional start codon AUG is inserted upstream of the orf7b start codon. Thus, ORF7b may be expressed by “leaky scanning” of the ribosome (Liu et al., 2014). The Golgi-restricted localization of ORF7b was attributed to the transmembrane domain (Liu et al., 2014). However, the role of ORF7a and ORF7b during the replication of SARS-CoV remains uncertain (McBride & Fielding, 2012).

### ORF8 protein

Human SARS-CoV-2 isolated from early patients, Civet SARS-CoV and other bat SARSr-CoV contains the full-length ORF8 (Fuk-Woo Chan et al., 2020). Two genomes of genus *Betacoronavirus* isolated from horseshoe bat, SARS-Rf-BatCoV YNLF_31C and YNLF_34C are 93% identical to the human and civet SARS-CoV genome. However, all Human SARS-CoV isolates from mid and late-phase patients contain a signature 29 nucleotide deletion in the orf8 gene, splitting it into orf8a and orf8b. This suggests that ORF8 may play a role in interspecies transmission. The generation of civet SARSr-CoV could be a result of a potential recombination event between SARSr-Rf-BatCoVs and SARSr-Rs-BatCoVs identified around ORF8 (Lau et al., 2015). Interestingly, the new SARS-CoV-2 ORF8 shares very less homology with the conserved ORF8 or ORF8b isolated from Human SARS-CoV or its related viruses, Civet SARS-CoV, Bat-CoV YNLF_31C andYNLF_34C. The ORF8 lacks any known functional domain or motif. SARS-CoV ORF8b has an aggregation motif VLVVL (75–79aa) which is known to activate NLRP3 inflammasomes and trigger intracellular stress pathways (Fuk-Woo Chan et al., 2020), but this is not conserved in SARS-CoV-2. Notably, both ORF3 and ORF8 in SARS-CoV-2 are highly divergent from the interferon antagonist ORF3a and inflammasome activator ORF8b in SARS-CoV (Yuen et al., 2020).

### ORF10 protein

The SARS-CoV-2 ORF10 protein has no homologs in SARS-CoV and any other members of genus *Betacoronavirus* (Xu et al., 2020). Viruses are known to sabotage ubiquitination pathways for replication and pathogenesis. The ORF10 interacts with specifically the CUL2^ZYG11B^ complex and the rest of the members of the Cullin-2 (CUL2) RING E3 ligase complex. ZYG11B degrades substrates with exposed N-terminal glycine residues. By studying the ORF10 interactome, it was observed that it interacts the most with the CUL^ZYG11B^ complex (Gordon et al., 2020). This evidence shows that ORF10 might bind to this complex and hijack its ubiquitination of restriction factors (Gordon et al., 2020).

## Methodology (genomes, databases, and tools used in this study)

Complete genomes, belonging to SARS-CoV-2 (167 genomes), SARS-CoV (312 genomes) and Pangolin CoV (five genomes) were downloaded from NCBI Virus webpage (https://www.ncbi.nlm.nih.gov/labs/virus/) as on 29 March 2020. Similarly, RefSeq assemblies of 56 suborder Cornidovirineae genomes (including 18 from *Betacoronavirus* genus) along with the genome of Paguronivirus-1 (as an outgroup) were downloaded as on 1 April 2020. The latest version of the Virus database was downloaded from the NCBI Virus page as on 6 April 2020.

For alignments of the studied genomes, as required for phylogeny and mutational studies, we have used MUSCLE v3.8.1551 and MEGAX (Edgar, 2004; Kumar et al., 2018) tools as per their default settings. To generate maximum likelihood phylogenies of genome-based alignments, RAxML v8.2.12 (Stamatakis, 2014) was used to perform rapid bootstrap analysis and search for best­scoring ML tree in one program run (“-f a” parameter) using GTRGAMMA as nucleotide substitution model (-m) with 100 bootstrap values. After obtaining the newik trees from RAxML, an online iTOL platform (Letunic & Bork, 2019) was used to visualize phylogenetic trees as shown in this study. For each experiment in respective result sections, we have provided comprehensive details about the genomes under study, research methodology, and used parameters.

For the pangenome study, Proteinortho v6.0.12 (Lechner et al., 2011) was used with an E value cutoff 1E−05 for each identified hit along with other default settings. It utilizes diamond v0.8.36.98 and BLAST 2.8.1+ (Christiam et al., 2009; Buchfink, Xie & Huson, 2014) to identify ortholog proteins using reciprocal blast strategy. For the identification of pairwise average nucleotide identity (ANI) values within all genomes under study, PYANI v0.1.2 (Pritchard et al., 2016) program was used. Throughout this study, the Wuhan Hu-1 strain has been used as a reference for SARS-CoV-2 genomes.

## Results and Discussion

### Human SARS-CoV-2 genome is quite disparate as compared to other *Betacoronavirus* genomes

The SARS outbreak in 2003 and MERS in 2012 had already set a precedent for widespread genome analysis, therefore, with the recent emergence of COVID-19 in November 2019, extensive sequencing and genome analysis of SARS-CoV-2 isolates are being performed. Since February 2020, several Bat and Pangolin coronaviruses have also been sequenced to understand the probable origin of SARS-CoV-2. In this study, we have analyzed 312 SARS-CoV, 167 SARS-CoV-2 and 5 Pangolin CoV genomes to understand their genomic conservation, unique genes, mutational hotspots and respective evolution and origin.

### Phylogeny relationship at suborder Cornidovirineae level

SARS-CoV-2 is a member of suborder *Cornidovirineae*, family *Coronaviridae*, genus *Betacoronavirus*, and subgenus *Sarbecovirus*. In this study, we have tried to understand the strain diversity and evolutionary relationships at different taxonomy levels in a top-bottom classification scheme. For this analysis, the complete whole-genome sequences of 56 suborder *Cornidovirineae* reference genomes were analyzed, which included members from five genera i.e. 23 *Alphacoronavirus*, 20 *Betacoronavirus*, 10 *Deltacoronavirus* and three *Gammacoronavirus* and the genome of Paguronivirus-1 was included as an outgroup for this study. Their genome length varied from 25,425-31,686 nucleotides. ClustalW alignment (using MEGA-X) generated an alignment of 35,601 nucleotides off which 17,284 conserved sites were stripped and used to generate an ML-based phylogeny using RAxML. We noted that this phylogeny is unambiguously able to demarcate all the genera into their respective monophyletic clades (Fig. S1). The significant variations amongst branch length distances indicate the relative diversity within each genus.

### Phylogeny relationship at genus *Betacoronavirus* level

We generated the phylogeny of 18 *Betacoronavirus* reference strains (along with five Pangolin CoV strains) using their complete genomes ranging within 29,114–31,526 nucleotides. ClustalW based whole genome alignment using MEGAX generated an alignment of length 33,346 from which 24,555 conserved nucleotide sites were stripped and used to generate ML phylogeny (Fig. S2). Three distinct clades were seen in the phylogeny: the first clade includes strains from subgenus *Sarbecovirus*, *Nobecovirus* and *Hibecovirus*, second with *Embecovirus* members, and the third clade of members of subgenus *Merbecovirus*. Within the first clade, both members of subgenus *Sarbecovirus* (SARS-CoV and SARS-CoV-2) are grouped with Pangolin CoV strains, which is supported by their percentage identity. All Pangolin CoV genomes are >99.8% identical to each other whereas their closest relatives are the SARS-CoV-2 reference strain (~85% identity), followed by the SARS-CoV reference strain (~79% identity). These three groups are closest (~57% identity) to their sister branch occupied by subgenus *Hibecovirus* strain: Bat Hp-betacoronavirus Zhejiang 2013. Two reference strains of another subgenus *Nobecovirus* (~67% identity with each other) form a separate clade that is closest (~52% identity with other group members) to the above-mentioned clade comprising subgenus *Sarbecovirus* and *Hibecovirus*. The second clade only has representatives of subgenus *Embecovirus* including Murine hepatitis virus, Bovine CoV, Rabbit CoV, Rat CoV, etc. Some of these strains form respective subclades in the phylogeny based on their closeness. Strains belonging to subgenus *Merbecovirus* that include the MERS, form the third clade. MERS genomes (England 1 and MERS Co.) are 99.7% identical to each other and therefore form a separate sub-clade. As observed from their phylogenetic branch lengths and percentage identity matrix, all subgenera are quite distinct from each other. As expected, members of each subgenus are closer to each other, therefore forming respective monophyletic clades.

### Phylogeny relationship at subgenus *Sarbecovirus* level

**Figure 1:**
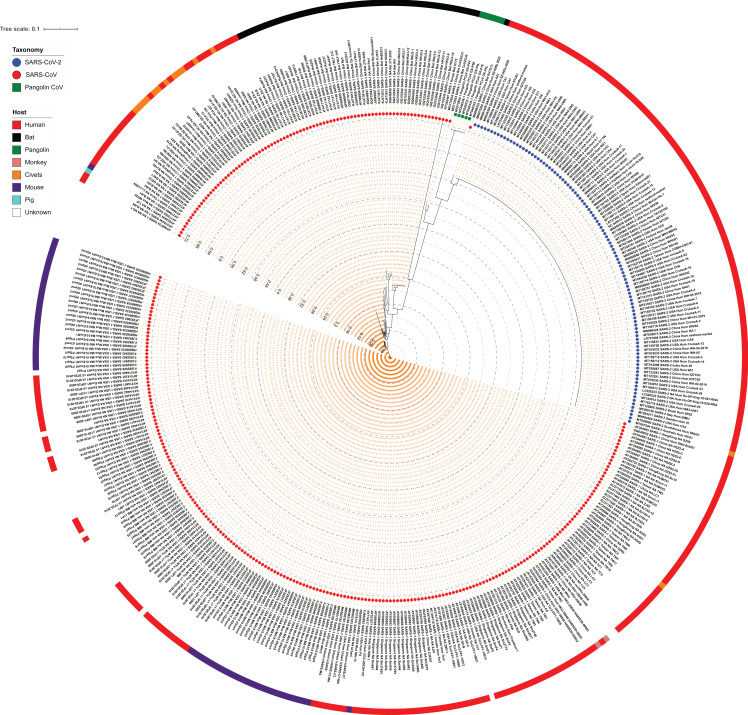
Maximum Likelihood (ML) phylogeny representing relationship amongst 312 SARS-CoV, 103 SARS-CoV-2, and five Pangolin CoV strains. The whole-genome sequences of 420 isolates were aligned using MUSCLE and stripped to include the highly conserved alignments across all strains. The final alignment was subjected to RAxML to generate the ML phylogeny utilizing the GTRGAMMA model of nucleotide substitution with 100 bootstrap replicates. The phylogeny is depicted with branch length consideration. The inner-circle represents the taxonomy of all strains (depicting SARS-CoV, SARS-CoV-2 and Pangolin CoV). The outermost circle represents the respective host of each strain. Inner Blue and Gray alternative dashed lines represent an internal tree scale with a branch length increment of 0.04 from inside to outside.

To understand the similarities and differences among the strains of subgenus *Sarbecovirus*, we analyzed the complete genomes of 103 SARS-CoV-2 and 312 SARS-CoV isolates including the reference strains of Wuhan Hu-1 (SARS-CoV-2) and Tor2 (SARS-CoV). Since Pangolin CoV belongs to subgenus *Sarbecovirus*, we also included 5 Pangolin CoV genome sequences in our dataset and aligned them using MUSCLE. The genome size of SARS-CoV, Pangolin CoV and SARS-CoV-2 strains were in the range of 29,013–30,311 nucleotides, 29,795–29,806 nucleotides and 29,325–29,945 nucleotides, respectively. The alignment of length 31,718 nucleotides was stripped to a conserved region of 26,762 nucleotides (sites with gaps in between them were excluded) which was used to generate a maximum likelihood phylogeny using RAxML (Fig. 1; Fig. S3). We noted from the phylogeny that SARS-CoV-2 strains form a single monophyletic clade as compared to the SARS-CoV. SARS-CoV strain RaTG13 procured from the bat in 2013 from Yunnan, China is closest to SARS-CoV-2 strains, suggesting that they might have diverged from a common ancestor. Average Nucleotide Identity (ANI) studies also indicated that the SARS-CoV-2 reference genome and the Bat RaTG13 branch share 96.11% identity at the genome level, a finding supported by earlier reports (Lv et al., 2020; Zhou et al., 2020). The next closest strains in the phylogeny were those of Pangolin CoV, all forming a subclade. Other close relatives are the SARS-CoV strains Bat CoVZC45 and Bat CoVZXC21, procured from Zhoushan, eastern China in 2018. The phylogeny suggests that Pangolin CoVs are closer to SARS-CoV-2 strains as compared to CoVZC45 and CoVZXC21, however, genome-wide percentage identity suggests the opposite—the genomes of both CoVZC45 and CoVZXC21 are ~89% identical to SARS-CoV-2, as opposed to its 86% identity with Pangolin CoVs.

We also examined the ANI scores of the above-identified closest strains of SARS-CoV-2 with all SARS-CoV, SARS-CoV-2 and Pangolin CoV genomic sequences used in this study (Table S2). We found that the Bat RaTG13 strain is 95.97–96.12% identical when compared to all SARS-CoV-2 genomes under study. Amongst the SARS-CoV strains, Bat CoVZC45 and Bat CoVZXC21 are its closest neighbors with 89% genome identity, indicating a common ancestor in bat coronaviruses, whereas, with Pangolin CoV, they share lowest (~86%) nucleotide identity. Bat CoVZC45 and Bat CoVZXC21 are ~97% identical to each other, whereas their identity with SARS-CoV-2 (~89%) is higher than that with SARS-CoV (86–89%) and lowest with Pangolin CoV (~85%). Similarly, Pangolin CoVs share maximum identity with Bat RaTG13 SARS-CoV (86.50%) followed by SARS-CoV-2 (86.30–86.38%) and lowest with other SARS-CoV. Therefore, we can argue that as SARS-CoV-2, Bat RaTG13 and Pangolin CoV have considerable genome similarity, they possibly diverged from a common ancestor and SARS-CoV-2 was later transmitted to humans through recombination and transformation events via an unknown intermediate host.

### Phylogeny relationship at SARS-CoV-2 level

**Figure 2:**
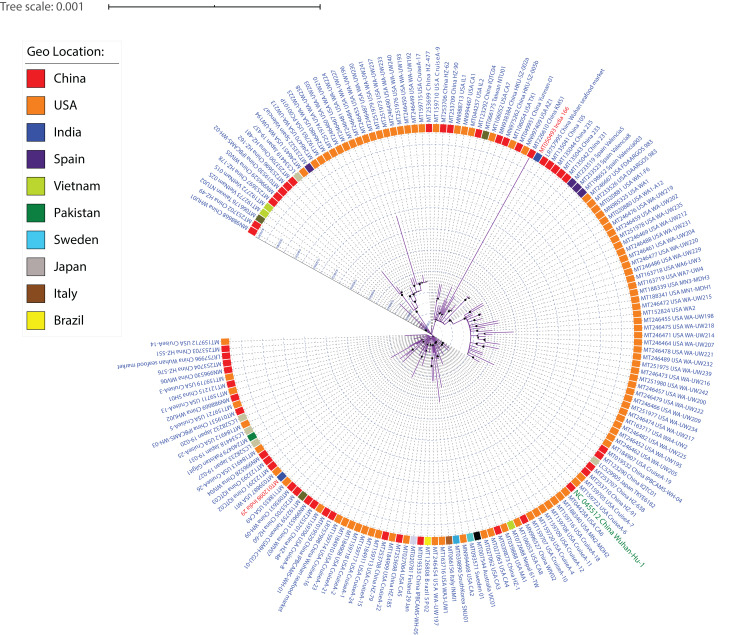
ML phylogeny representing relationship amongst 167 SARS-CoV-2 strains. The whole-genome sequences of 167 isolates were aligned using MUSCLE, stripped to include only conserved alignments, and subjected to RAxML to generate the ML phylogeny utilizing the GTRGAMMA model of nucleotide substitution with 100 bootstrap replicates. The phylogeny is depicted with branch length consideration. The inner circle represents the respective geo-location of each strain. Inner Blue and Gray alternative dashed lines represent an internal tree scale with a branch length increment of 0.00005 from inside to outside.

SARS-CoV-2 strains, like other viruses, have a high mutation rate for better adaptability and survival. Therefore, one of our aims was to understand the diversity among SARS-CoV-2 isolates. Whole-genome sequences of 167 SARS-CoV-2 were aligned using MUSCLE (alignment length 29,950 nucleotides) and the conserved sites of length 29,725 were used to generate a maximum-likelihood phylogeny using RAxML (Fig. 2; Fig. S4). Irrespective of high similarity amongst all (>99.90% identity), several subclades can be identified from the phylogeny based on their distance from each other, depicting their subtypes. Our data has 97 isolates from the USA, 46 from China, five from Japan, four from Spain, three from Taiwan, two from Vietnam and India, one isolate each from Sweden, South Korea, Pakistan, Nepal, Italy, Finland, Brazil and Australia. From the genome sequence data of 15 distinct geographical locations, we were able to identify a few phylogenetic clusters depicting country-specific patterns. We identified multiple clusters per country suggesting the existence of multiple subtypes. The KMS1 isolate from China was found to be the most distinct one, followed by WA-UW230, WA-UW194, WA-UW218 isolates from the USA. The Wuhan Hu-1 isolate shares a sister clade with the USA Cruise samples, indicative of high similarity. Isolates sampled at the University of Washington (WA, USA) form two separate subclades within two different clades in the phylogeny, suggesting that even amongst people residing in a limited area in the state of Washington, multiple strains exist and are causing COVID-19. Furthermore, both the WA subclades have Valencia-Spain isolates as the closest branch/subclade, suggesting that at least two individuals from these two locations independently encountered each other and transmitted different subtypes of the virus. This study also included two distinct genomes sampled from India which were distributed by the phylogeny into two separate clusters signifying the existence of different subtypes.

As strains from diverse locations continue to be sequenced and analyzed, more reliable country-specific patterns showing relatedness can be obtained. Similar to several cutting edge studies (Woo et al., 2010; Fuk-Woo Chan et al., 2020; Zhang, Wu & Zhang, 2020; Rehman et al., 2020), this study also provides an example of how phylogenetic analysis can help generate clusters of identical strains, further enabling the identification of signature mutations amongst those diverse groups.

### Mutations are continuously diversifying the Human SARS-CoV-2 strains

**Figure 3:**
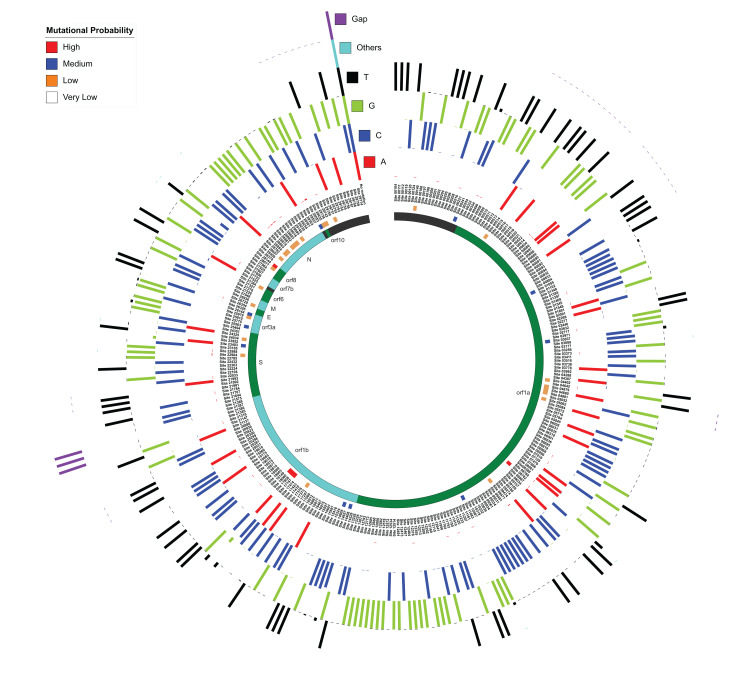
Mutation sites and hotspots distribution amongst 167 SARS-CoV-2 genomes as compared to Wuhan Hu-1. MUSCLE-based alignment was used to analyze the distribution of all nucleotides (including gap and other nucleotides) at each alignment site and the relative percentage of each nucleotide is represented here in a circular manner respectively for each nucleotide. The innermost circle represents the respective orf and non-coding DNA according to the location as mentioned in the third circle from inside. The second circle represents the mutational probability score of each locus distributed in high, medium, low and very low categories.

Like the previous section, the whole genome sequence alignment of 167 SARS-CoV-2 isolates were used for this study. As anticipated, ~100 nucleotides at both N and C-terminals were highly diverse and, therefore, changes in these sites were not considered in this study. For the rest of the alignment, the percentage of nucleotide occurrence at each site (along with gap and other non-standard nucleotides) was calculated using the SARS-CoV-2 Wuhan Hu-1 genome as the reference (Fig. 3). The mutational probability was classified into four categories based on random distribution patterns: high (>19% variation), medium (4–19% variation), low (2–3% variation) and very low (<2% variation). Overall, 415 mutation sites were identified, among which non-standard nucleotides were present in the genome at 125 sites, which have been ignored in this study. We ultimately selected 290 sites with a confirmed variation. Amongst these, we found 43 sites with high, medium and low mutation probabilities within 9 out of 12 protein-coding regions in SARS-CoV-2 genomes, which also included the genes (S, M and N), identified as core proteins across genus *Betacoronavirus*. Amongst all mutation sites, we found 21 sites within the gene encoding Spike protein, 18 sites in the gene encoding Nucleocapsid protein and three sites in gene encoding M protein, suggesting that even these highly conserved protein-coding regions (core proteins as later identified via pan-genome analysis) are capable of mutating. Also, we recognized 142 mutational sites in orf1a, 50 in orf1b, 6 in orf3a, 2 in the envelope protein-coding gene, 4 in orf6, and orf8, 3 in orf7b and 1 in orf10. We also identified 36 sites within non-coding regions.

Amongst sites with high mutation probability, C8,782T in orf1a showed ~35% variation from C to T, suggesting a transition mutation. Likewise, T28,144C in orf8 also has ~35% transition mutation variations. We found three hotspot mutational sites within the orf1b region of orf1ab, which has three transitions, two C to T (C17,747T and C18,060T; ~20% variation) and one A to G (A17,858G; 19% variation). In the spike protein-coding region, we were able to identify one transition site (A23,403G nucleotide) with medium (11%) mutational probability. Overall, we were able to identify nine hotspots with more than two consecutive probable mutational nucleotides in vicinity (197–210 (non-coding), 508–522 (orf1a), 686–694 (orf1a), 4,879–4,881 (orf1a), 20,298–20,300 (orf1b), 21,385–21,389 (orf1b), 21,991–21,993 (S), 28,878–28,883 (N), 29,750–29,759 (non-coding)). Most of these hotspot sites showed ~1–2% mutational probabilities and none of these have high or medium mutational probability as discussed in Fig. 3. We believe these genomic locations are the probable sites of evolutionary divergence, and therefore govern the evolutionary capabilities of these viruses.

We also analyzed transition and transversion possibilities within all these mutational combinations. Out of the 290 identified mutational sites, 244 had a nucleotide substitution, distributed into 158 transitions and 86 transversions. Transition events are known to be more frequent and less likely to cause a change at the protein level as compared to transversions (Sanjuán et al., 2010). Most transition events are silent mutations, while transversion events may cause change at the protein level and therefore impact the pathogenesis of the disease (Lyons & Lauring, 2017). Therefore, we suggest that amongst SARS-CoV-2 genomes, transversion events, that are occurring in significant numbers, would provide more evolutionary diversity and possibly lead to divergence, as compared to transition events.

### Evolutionary relationship amongst SARS-CoV-2, Bat CoV and Pangolin CoV

**Figure 4:**
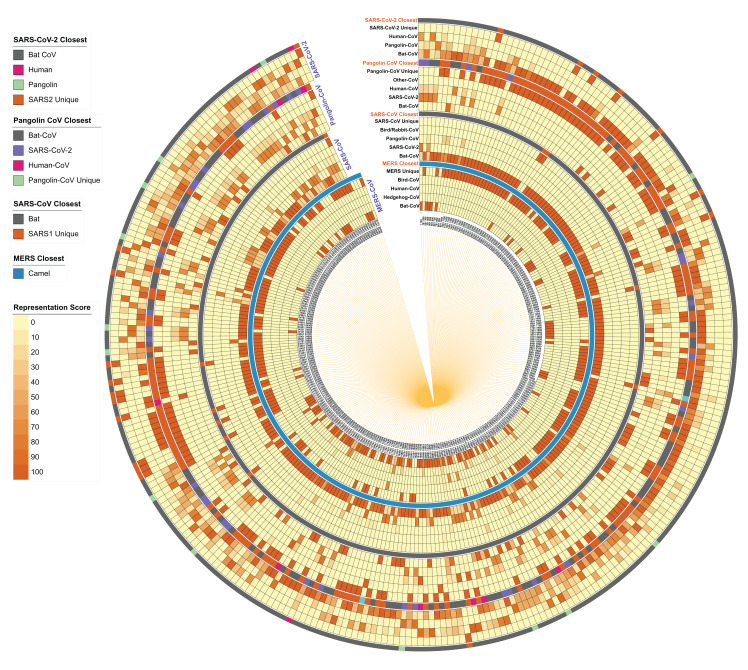
Host distribution of each 100-nucleotide segment in the reference genomes of MERS, SARS-CoV, Pangolin CoV, and SARS-CoV-2. Each 100-nucleotide segment per strain was subjected to BLASTn against the virus database. Self-category hits were removed from the analysis, host information of top 50 hits was identified, and the relative percentage is represented here as a heatmap for each segment in each genome. In the closest category, the host information of best hit (after removing self-hit) is represented in the form of a multi-color stripe. The innermost circle represents the location of each segment to start from 1 to 100 nucleotides to the total genome length of the genome.

To re-examine the closest hosts of these viruses, we performed homology studies on small genome fragments (100 nucleotides) to identify their close relatives. We segmented each of the SARS-CoV-2, Pangolin CoV, SARS-CoV and MERS genome (reference genome of each category) into 100 nucleotide fragments, identified their closest homologs in the virus database, filtered the strains belonging to its taxonomy, selected top 50 hits per segment, calculated the host CoV distribution and represented it as a heatmap for each segment/strain along with the closest (topmost) hit per segment/strain (Fig. 4). MERS-CoV is already known to originate from the bat, however, the infection spread via camels either directly/indirectly (Azhar et al., 2014). On excluding Camel CoVs from the data, we found several segments showing maximum similarity representation in Bat CoVs, followed by Hedgehog and Human CoV strains, however, most of the parts were uniquely present in MERS. As expected, when Camel CoV strains were included, they were the closest best hit as indicated in the “MERS Closest” category (Fig. 4).

When we further investigated the same patterns in the SARS-CoV genome, we identified the Bat CoV as the closest strain for most of the reference segments, followed by SARS-CoV-2 and Pangolin CoV. In the closest category, we found Bat CoV as the closest suggesting their evolutionary origin. However, many segments do not show any closeness with any strain, indicating their uniqueness in the reference genome. We performed the same analysis on the Pangolin CoV strain GX-P5E and found that several segments have close homology-distribution within SARS-CoV and SARS-CoV-2 genomes. Interestingly, as visible in the “Pangolin CoV Closest” category, several hits show maximum similarity with Bat SARS-CoV, followed by SARS-CoV-2 and a few Human CoVs.

Likewise, SARS-CoV-2 genome fragments show maximum homology representation in Bat CoV followed by Pangolin CoV. In the “closest category”, Bat CoV indisputably stands out as the closest host, which is corroborated by whole-genome phylogeny and the percentage identity matrix studies. However, the presence of several Pangolin CoV homologous segments in this analysis indicates that certain specific segments in the SARS-CoV-2 genome share a closer relationship with Pangolin CoV strains as compared to Bat CoVs.

Overall, our analysis indicates that the SARS-CoV-2 genome has a close relationship with Bat CoV and Pangolin CoV, along with the presence of a few unique segments. This reasserts that SARS-CoV-2 strains might have evolved from a common ancestor of Bat SARS-CoV and Pangolin CoV strains and during evolution, several segments in the SARS-CoV-2 genome might have diverged as compared to Bat SARS-CoV and Pangolin CoV. The recognized unique genomic segments in this study may be used as potential targets for diagnostic kits.

### Pan-genome (Core, accessory and unique gene) analysis

**Figure 5:**
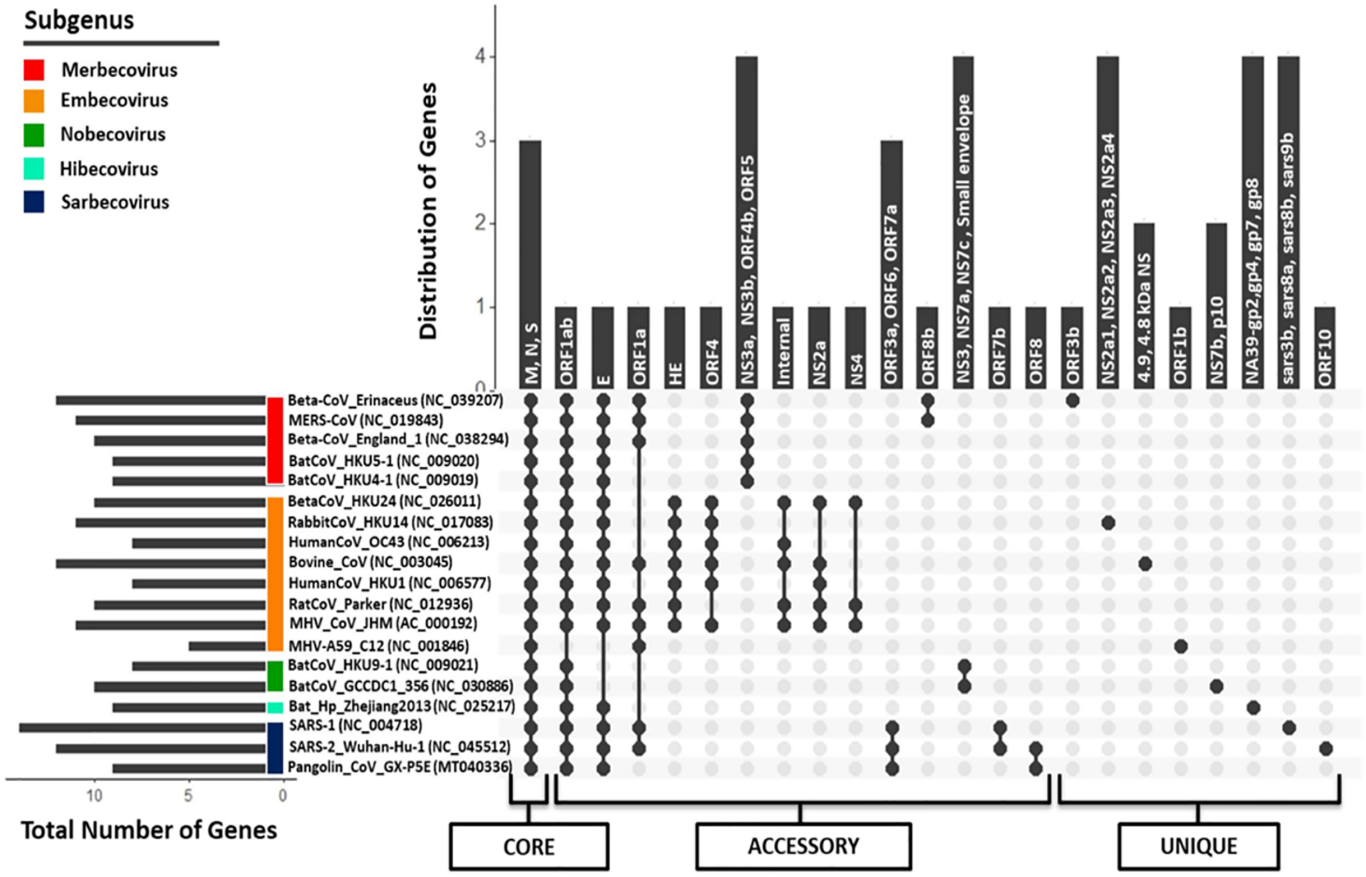
Pan-genome (Core, accessory, and unique) analysis amongst 19 *Betacoronavirus* reference genomes (including one Pangolin CoV). The *X*-axis represents the strain combinations as black-filled circles, whereas *Y*-axis represents the distribution of genes in the respective combinations and inside each vertical bar, those gene names are written. For each strain, their respective subgenus category has been pointed out on the left side as the colored scale in the corner. In the left-down corner, the number of genes encoded by each genome is depicted as a horizontal bar. In the lowermost panel, pan-genome categories (core, accessory and unique genes) are shown.

Regardless of their approximately similar genome size, the members of *Betacoronavirus* harbor huge diversity (5–14 CDS) in the number of protein-coding regions, as per the available NCBI genome annotations. SARS-CoV and SARS-CoV-2, belonging to subgenus *Sarbecovirus*, have 14 and 12 protein-coding regions respectively, revealing the diversity within the same subgenus. Similarly, Pangolin CoV (studied strain: GX-P5E), which also belongs to subgenus *Sarbecovirus*, has only nine protein-coding regions. To identify the presence of (co-)orthologous proteins present across all these genomes, a bidirectional (reciprocal) best BLAST hit-based homology detection strategy was followed using the tool Proteinortho (Lechner et al., 2011) (Fig. 5; Fig. S5). A total of 44 protein-coding region clusters represent the pan-genome of nineteen studied genus *Betacoronavirus* members amongst which, 3 protein-coding regions (S, M and N proteins) were found to be conserved across all genomes, representing the core genome. A similar analysis has been performed on all *Betacoronavirus* species; however, it does not include Pangolin CoV (Alam et al., 2020). Our study also recognized that the pan-genome of genus *Betacoronavirus* is open, which means that with the addition of a new species, the pan-genome is continuously increasing (Fig. S6). The increase in the pan-genome at each step is approximately proportional to the increase in unique genes. Since each newly identified strain has additional unique gene sequences, it is effectively impossible to predict their complete pan-genome. As expected, the members of each subgenus, which are closest to each other, have only a few variations, however, with the addition of a new subgenus member(s), the pan-genome expands immediately.

orf1ab, the largest coding region, codes for two polyproteins ORF1a and ORF1ab (266–13,468,13,468–21,555) because of the “leaky mechanism” of the ribosome, a (-1) frameshift just upstream of the orf1a translation termination codon. All CoVs, except for strain MHV A59 of subgenus *Embecovirus*, translate the full-length orf1ab. MHV A59, however, has separate polyproteins from orf1a and orf1b. Due to the absence of ribosomal slippage reported in several coronaviruses, *ORF1a* is encoded by only 9 out of 18 genomes, making it an accessory protein cluster of the pan-genome. *ORF1b* polyprotein is encoded as a part of orf1ab in most of the species belonging to genus *Betacoronavirus*, again except for strain MHV A59, where it is encoded separately as RdRp, forming a unique gene. *Envelope protein* (76–82 aa across *Betacoronavirus*) is another accessory protein encoded by 15 out of 18 genomes. In another cluster, we identified the presence of *small envelope protein* in two out of those three genomes where E protein is absent (both in subgenus *Nobecovirus*). No E protein-encoding region is found in the MHV A59 genome annotations. The gene encoding *ORF3* is distributed into two subunits (orf3a and orf3b) in SARS-CoV (via an internal ribosomal entry mechanism), whereas only the first subunit (orf3a; 276 aa) is present in SARS-CoV-2. The gene for ORF3a is conserved only amongst subgenus *Sarbecovirus* organisms. This protein activates the pro-transcription of gene IL-1β and helps its maturation; both signals are prerequisites for NLRP3 (NOD-, LRR- and pyrin domain-containing protein 3) inflammasome activation during infection (Siu et al., 2019). Conservation of ORF3a across SARS-CoV-2, SARS-CoV and Pangolin CoVs provides a clue about its unique infection strategy. *ORF3b* (155 aa) is only present in SARS-CoV and absent in all other coronaviruses. It has been suggested that ORF3b supports SARS-CoV infection by inhibiting the type-1 Interferon (IFN) synthesis and therefore, IFN signaling (McBride & Fielding, 2012). *ORF6* is also a subgenus *Sarbecovirus* specific protein (conserved across SARS-CoV, SARS-CoV-2 and Pangolin CoV) localized in the Endoplasmic Reticulum or the Golgi membrane. During infection, it interacts with and interrupts the nuclear import complex formation. During infection-caused interferon signaling, this interruption inhibits STAT1 transportation to the nucleus and blocks the expression of genes involved, therefore mimicking an antiviral state (Frieman et al., 2007).

Another accessory protein of the *Betacoronavirus* pan-genome is *ORF7a*, which is a unique protein amongst subgenus *Sarbecovirus* members. In SARS-CoV, it is known that bone marrow stromal antigen-2 (BST-2 or tetherin or CD317) can restrict the virus release from inside the cell causing partial inhibition of infection. However, ORF7a can inhibit the action of this protein, thus promoting the infection (Taylor et al., 2015). *ORF7b* is present in both SARS-CoV-2 and SARS-CoV but absent in Pangolin CoV NCBI annotations. Like orf7b in SARS-CoV, its initiation codon overlaps with orf7a via ribosome leaky scanning and it works as a structural component (Schaecher, Mackenzie & Pekosz, 2007). *orf8* gene (365 nucleotides) is uniquely present in SARS-CoV-2 and Pangolin CoV but absent in SARS-CoV. Distinctively, orf8 in SARS-CoV is separated into two proteins ORF8a (119 nucleotides) and ORF8b (254 nucleotides). ORF8 in SARS-CoV-2 is highly divergent from the inflammasome activator ORF8b in SARS-CoV (Yuen et al., 2020). The function of this protein is not clear yet, but it has been suggested that overall ORF8ab or ORF8a and ORF8b together modulate viral replication and pathogenesis in unknown fashion (McBride & Fielding, 2012). *ORF10* is a unique protein in SARS-CoV-2 strains. We also performed the homology analysis of ORF10 protein against the NCBI Non-redundant database and found no hits, which suggests that this protein is specific to SARS-CoV-2.

This analysis also revealed that some proteins are specific to a subgenus. Subgenus *Embecovirus* has a unique combination of proteins, namely HE, ORF4, internal protein, NS2a and NS3a, that are present in most genomes of this subgenus. These proteins might be considered unique to subgenus *Embecovirus* as compared to the rest of the genus *Betacoronavirus*. *Hemagglutinin Esterase* glycoprotein (HE) is a nonessential protein with sialic acid-binding and acetyl esterase activity that create tinier spikes (second attachment factor) on the viral envelope in several MHV strains. *orf4* (also annotated as orf5a, ns12.9) encodes a nonstructural accessory protein that is, known to function as type I interferon antagonist. The *internal* gene is encoded within the N gene in MHV CoV; however, its function is not well-known. Similarly, subgenus *Merbecovirus* members also have some unique proteins (NS3a, NS3b, NS4b, ORF5), that are absent in other subgenera. We also identified unique proteins in each organism, which were absent in other *Betacoronavirus*. Overall, it can be argued that although genus *Betacoronavirus* members have ~10 proteins coding regions per genome, they have conserved similarity in only ~10% (3–4 proteins) of the genes and their diversity (~40% accessory genes and ~50% unique genes) spans the rest of the genome, leading to their different pathogenesis across diverse hosts and distinct evolution. We identified that even among subgenus *Sarbecovirus* members (SARS-CoV, SARS-CoV-2 and Pangolin CoV), orf3b, orf7b, orf8, orf9b and orf10 are uniquely distributed within at least one genome, therefore depicting wide diversity within the same subgenus.

## Conclusions

Humanity has already encountered coronavirus infections twice in the past two decades, in the form of the SARS and MERS epidemics. The latest coronavirus infection, COVID-19, caused by the highly pathogenic and transmissible SARS-CoV-2, turned into a pandemic in sheer three months. This work provides a brief review of the taxonomy, history, origin, and genome organization of the virus, followed by comparative genomics research focused on understanding the evolutionary relationship amongst 167 SARS-CoV-2, 312 SARS-CoV and 5 Pangolin CoV genome sequences as available on 29 March 2020. We identified that SARS-CoV-2 strains form a monophyletic clade distinct from SARS-CoV and Pangolin CoV. This study reaffirmed that they are closest to the Bat CoV RaTG13 strain followed by Pangolin CoV, suggesting that SARS-CoV-2 evolved from a common ancestor putatively residing in bat or pangolin hosts. We identified several mutation sites and hotspots within SARS-CoV genomes with high, medium and low probabilities. Homology studies based on 100 nucleotide segments in the SARS-CoV-2 reference genome pointed out their close relationships with Bat and Pangolin CoVs, along with the unique nature of a few segments. We recognized that 44 protein-coding regions constitute the pan-genome for nineteen genus *Betacoronavirus* strains. Moreover, their pan-genome is open, highlighting the wide diversity provided by newly identified novel strains. Even members of subgenus *Sarbecovirus* are diverse relative to each other due to the relative presence of unique protein-coding regions orf3b, orf7b, orf8 and orf9b and orf10. Overall, this review and research highlight the diversity within the available SARS-CoV-2 genomes, their potential mutational sites hotspots, and probable evolutionary relationship with other coronaviruses, which might further assist in our understanding of their evolution, epidemiology and pathogenicity.

## Supplemental Information

10.7717/peerj.9576/supp-1 Supplemental Information 1 ML-based phylogeny of 56 family *Coronaviridae* members belonging to the genus *AlphaCoV*, *BetaCoV*, *DeltaCoV* and *GammaCoV*.

10.7717/peerj.9576/supp-2 Supplemental Information 2 ML-based phylogeny of 23 genus *Betacoronavirus* organisms (18 reference and 5 Pangolin CoV genomes) along with their percent identity analysis.

10.7717/peerj.9576/supp-3 Supplemental Information 3 Maximum Likelihood (ML) phylogeny representing relationship amongst 312 SARS-CoV, 103 SARS-CoV-2 and 5 Pangolin CoV strains:. The whole-genome sequences of 420 strains were aligned using MUSCLE which was stripped to include the highly conserved alignments across all strains. The final alignment was subjected to RAxML to generate the ML phylogeny utilizing the GTRGAMMA model of nucleotide substitution with 100 bootstrap replicates. The phylogeny is depicted without branch length consideration. The inner-circle represents the taxonomy of all strains (depicting SARS-CoV, SARS-CoV-2, and Pangolin CoV). The outermost circle represents the respective host of each strain. Inner Blue and Gray dashed lines represent an internal tree scale with a branch length increment of 0.04 from inside to outside.

10.7717/peerj.9576/supp-4 Supplemental Information 4 ML phylogeny representing relationship amongst 167 SARS-CoV-2 strains. The whole-genome sequences of 167 strains were aligned using MUSCLE, stripped to include only conserved alignments, and subjected to RAxML to generate the ML phylogeny utilizing the GTRGAMMA model of nucleotide substitution with 100 bootstrap replicates. The phylogeny is depicted without branch length consideration. The inner circle represents the respective geo-location of each strain. Inner Blue and Gray alternative dashed lines represent an internal tree scale with a branch length increment of 0.00005 from inside to outside.

10.7717/peerj.9576/supp-5 Supplemental Information 5 Pan-genome (Core, accessory, and unique) analysis amongst 19 *Betacoronavirus* reference genomes (including 1 Pangolin CoV). *Y*-axis represents the *Betacoronavirus* strains in the phylogenetic order (Fig. S2). For each strain, their respective subgenus category has been pointed out on the right side of phylogeny as the colored scale. The innermost circle represents pan-genome genes (44 in count) and gene names are also shown inside as text. For each strain, gene distribution as per the pan-genome is depicted.

10.7717/peerj.9576/supp-6 Supplemental Information 6 Correlation of the pan-genome of genus *Betacoronavirus* with the increasing numbers of strains suggests its open nature.

10.7717/peerj.9576/supp-7 Supplemental Information 7 SARS-CoV-2 taxonomy, research workflow, and methodology.

10.7717/peerj.9576/supp-8 Supplemental Information 8 Detailed information on strains used in this study.

10.7717/peerj.9576/supp-9 Supplemental Information 9 Genome-wide percentage identity matrix of phylogenetically closest strains of SARS-CoV-2 with all SARS-CoV, Pangolin CoV, and SARS-CoV-2 strains under study.

## Additional Information and Declarations

### Data Availability

The following information was supplied regarding data availability: The strains and genomes used in this research were retrieved from NCBI Virus (https://www.ncbi.nlm.nih.gov/labs/virus/vssi/#/) on 29 March 2020. This information is provided in Table S1.
