# Supplementary figures and images for: Understanding genomic diversity, pan-genome, and evolution of SARS-CoV-2
Source: PeerJ. 2020 Jul 17;8:e9576. doi: 10.7717/peerj.9576 (PMC7370936; doi:10.7717/peerj.9576)

Tree scale: 1

### Suborder Cornidovirineae

- Alpha CoV
- Beta CoV
- Delta CoV
- Gamma CoV

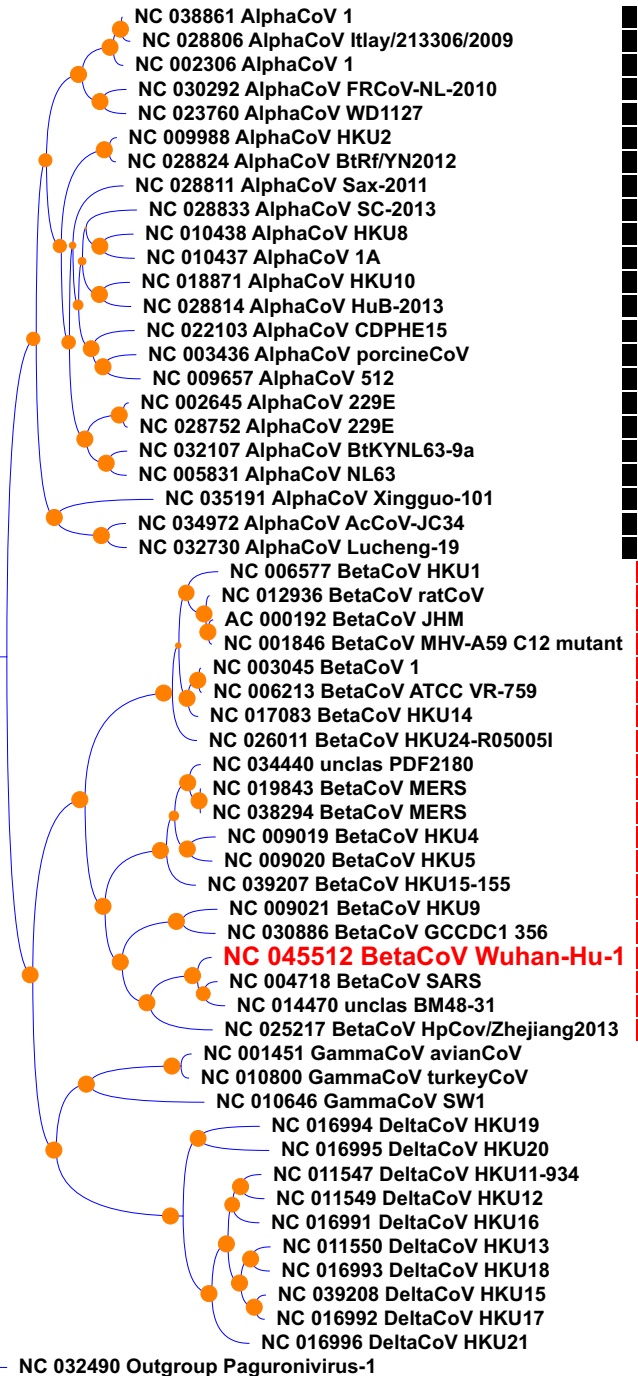

Supplement: Supplemental Information 1 [file peerj-08-9576-s001.pdf]

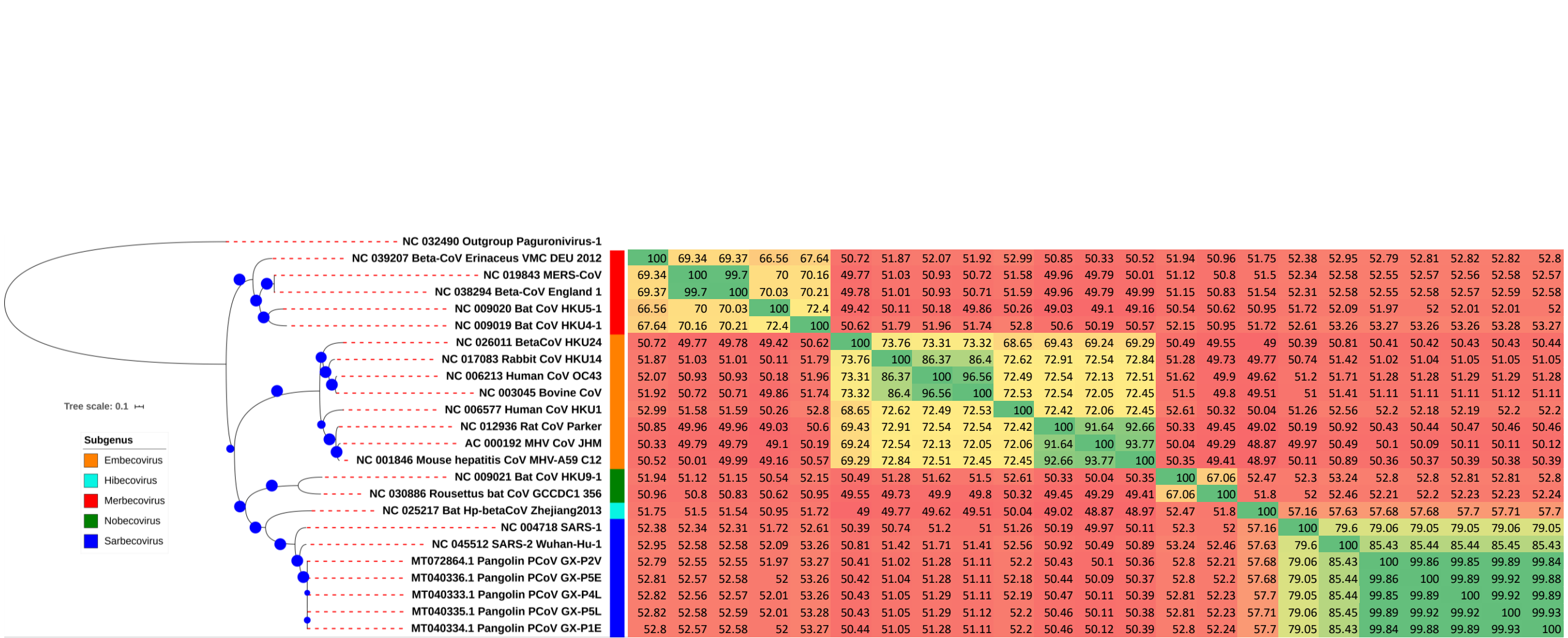

Supplement: Supplemental Information 2 [file peerj-08-9576-s002.pdf]

**Geo Location:**

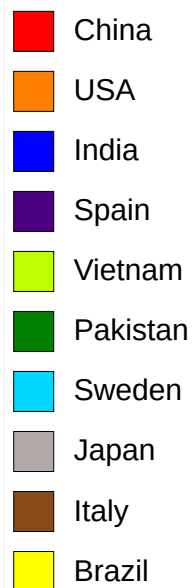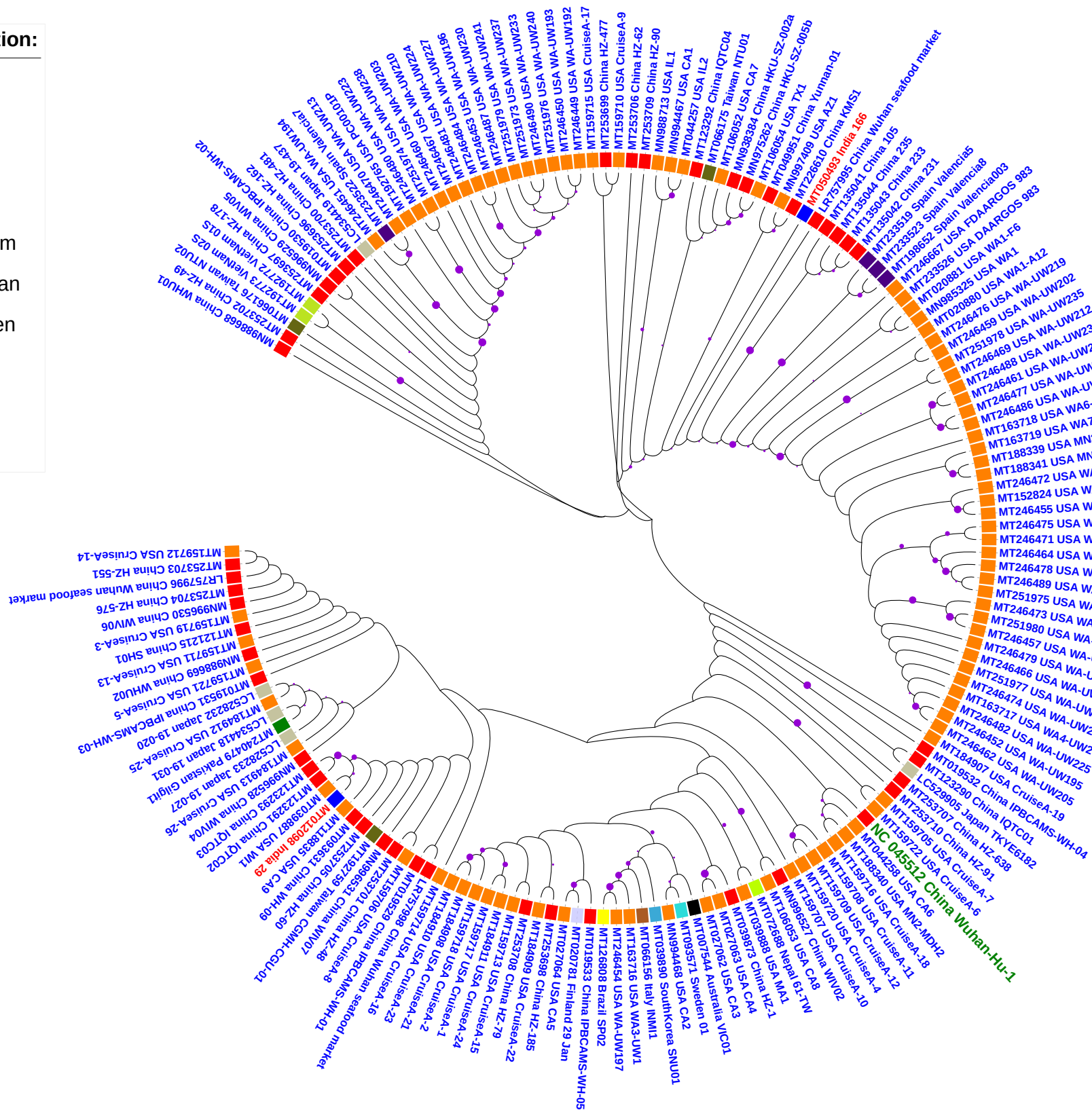

Supplement: Supplemental Information 4 — The whole-genome sequences of 167 strains were aligned using MUSCLE, stripped to include only conserved alignments, and subjected to RAxML to generate the ML phylogeny utilizing the GTRGAMMA model of nucleotide substitution with 100 bootstrap replicates. The phylogeny is depicted without branch length consideration. The inner circle represents the respective geo-location of each strain. Inner Blue and Gray alternative dashed lines represent an internal tree scale with a branch length increment of 0.00005 from inside to outside. [file peerj-08-9576-s004.pdf]

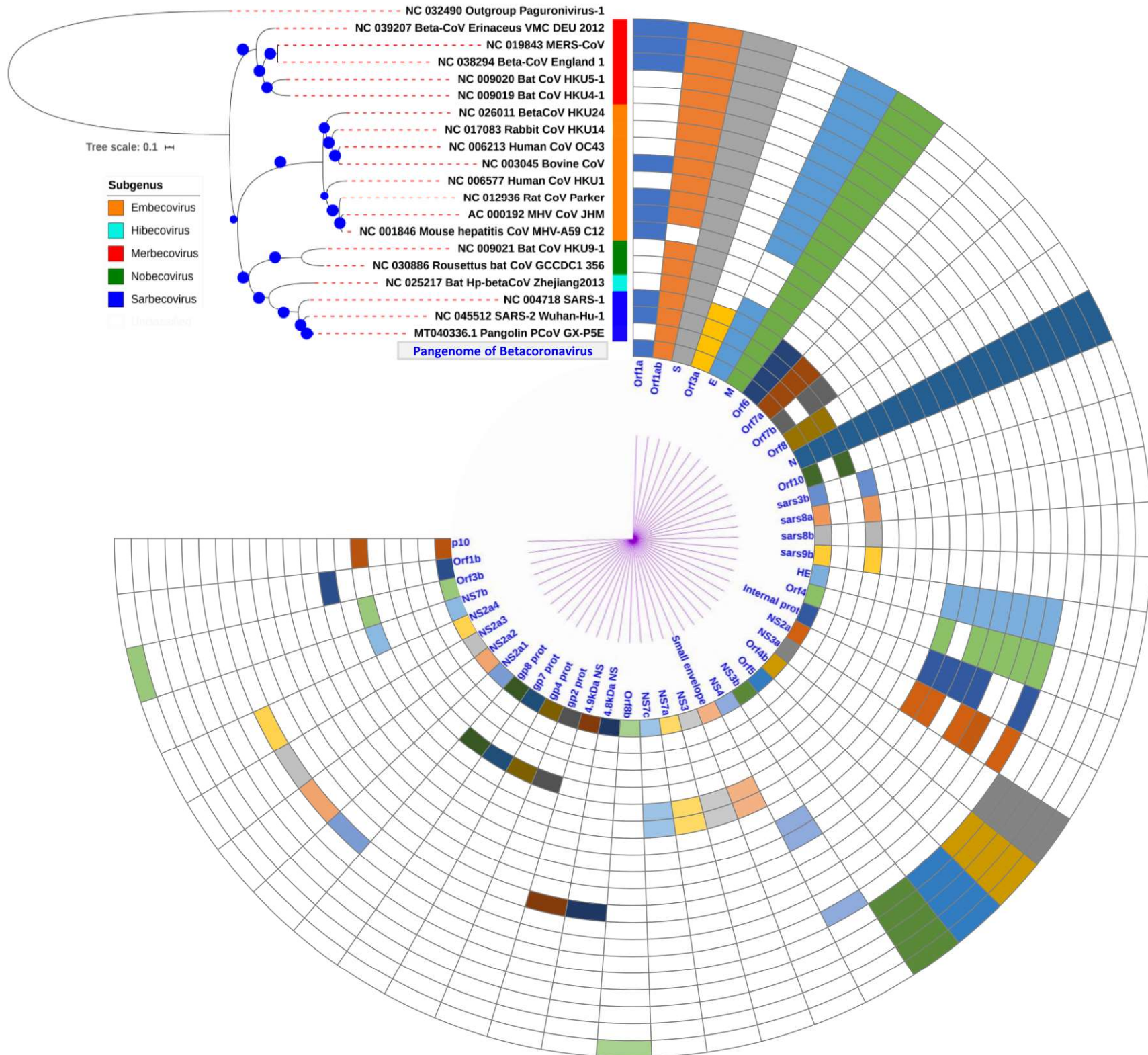

Supplement: Supplemental Information 5 — Y-axis represents the Betacoronavirus strains in the phylogenetic order (Fig. S2). For each strain, their respective subgenus category has been pointed out on the right side of phylogeny as the colored scale. The innermost circle represents pan-genome genes (44 in count) and gene names are also shown inside as text. For each strain, gene distribution as per the pan-genome is depicted. [file peerj-08-9576-s005.pdf]

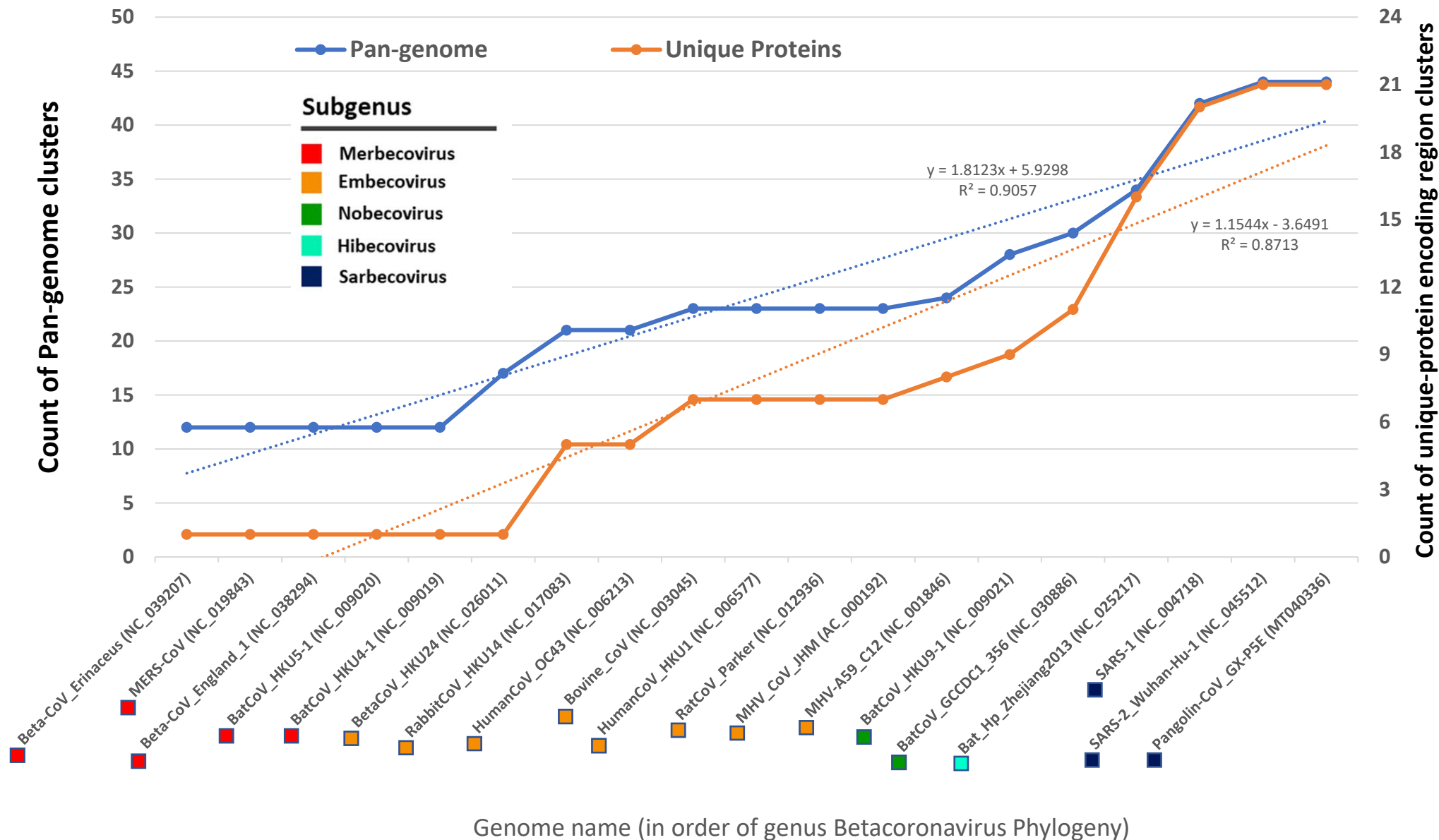

Supplement: Supplemental Information 6 [file peerj-08-9576-s006.pdf]
