# Supplementary material for: Understanding genomic diversity, pan-genome, and evolution of SARS-CoV-2
Source: PeerJ. 2020 Jul 17;8:e9576. doi: 10.7717/peerj.9576 (PMC7370936; doi:10.7717/peerj.9576)
Supplement: Supplemental Information 3 — The whole-genome sequences of 420 strains were aligned using MUSCLE which was stripped to include the highly conserved alignments across all strains. The final alignment was subjected to RAxML to generate the ML phylogeny utilizing the GTRGAMMA model of nucleotide substitution with 100 bootstrap replicates. The phylogeny is depicted without branch length consideration. The inner-circle represents the taxonomy of all strains (depicting SARS-CoV, SARS-CoV-2, and Pangolin CoV). The outermost circle represents the respective host of each strain. Inner Blue and Gray dashed lines represent an internal tree scale with a branch length increment of 0.04 from inside to outside. [file peerj-08-9576-s003.pdf]

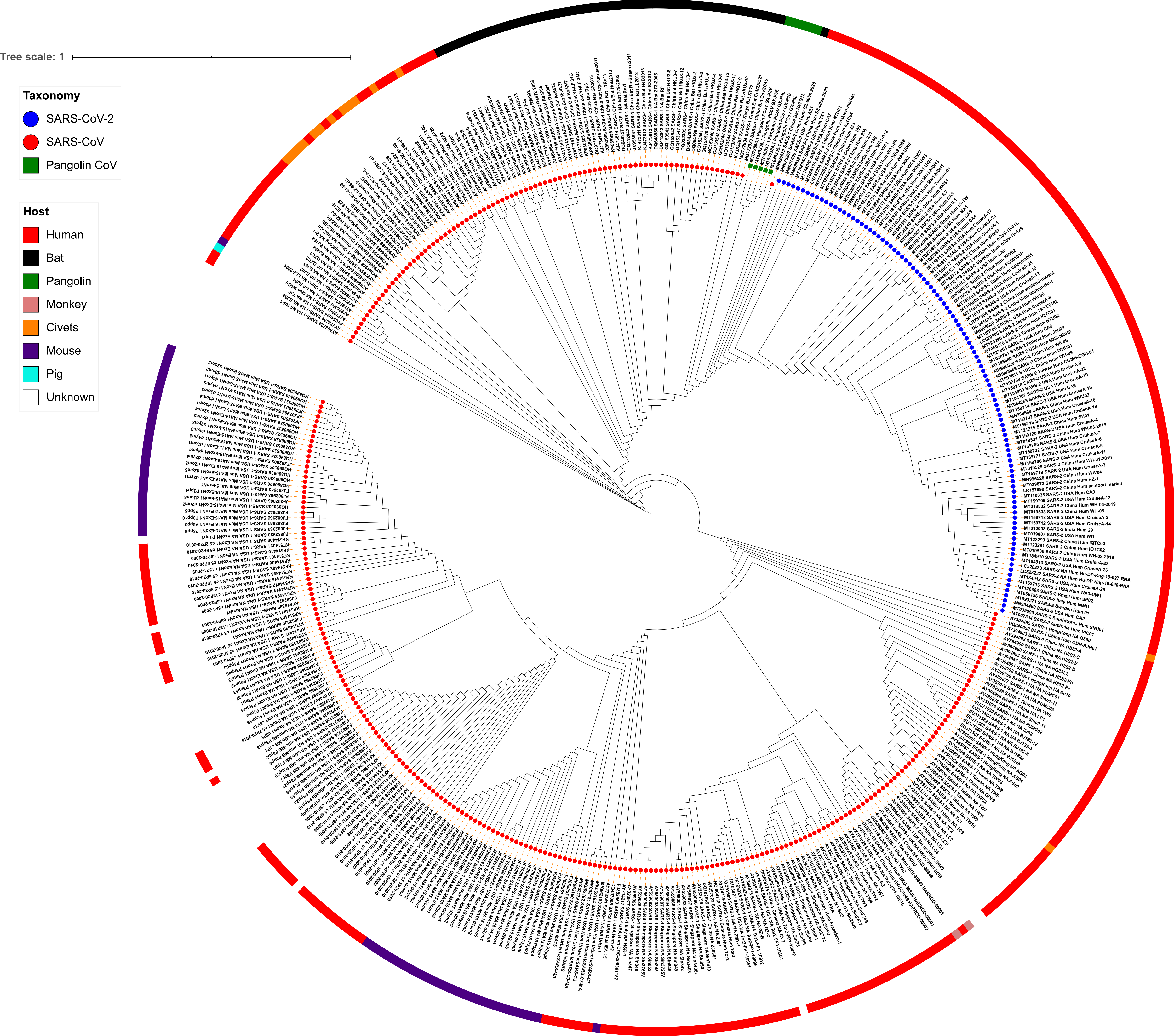
Tree scale: 1
Taxonomy
SARS-CoV-2
SARS-CoV
Pangolin CoV
Host
Human
Bat
Pangolin
Monkey
Civets
Mouse
Pig
Unknown
