# Supplementary material for: Understanding genomic diversity, pan-genome, and evolution of SARS-CoV-2
Source: PeerJ. 2020 Jul 17;8:e9576. doi: 10.7717/peerj.9576 (PMC7370936; doi:10.7717/peerj.9576)
Supplement: Supplemental Information 7 [file peerj-08-9576-s007.pdf]

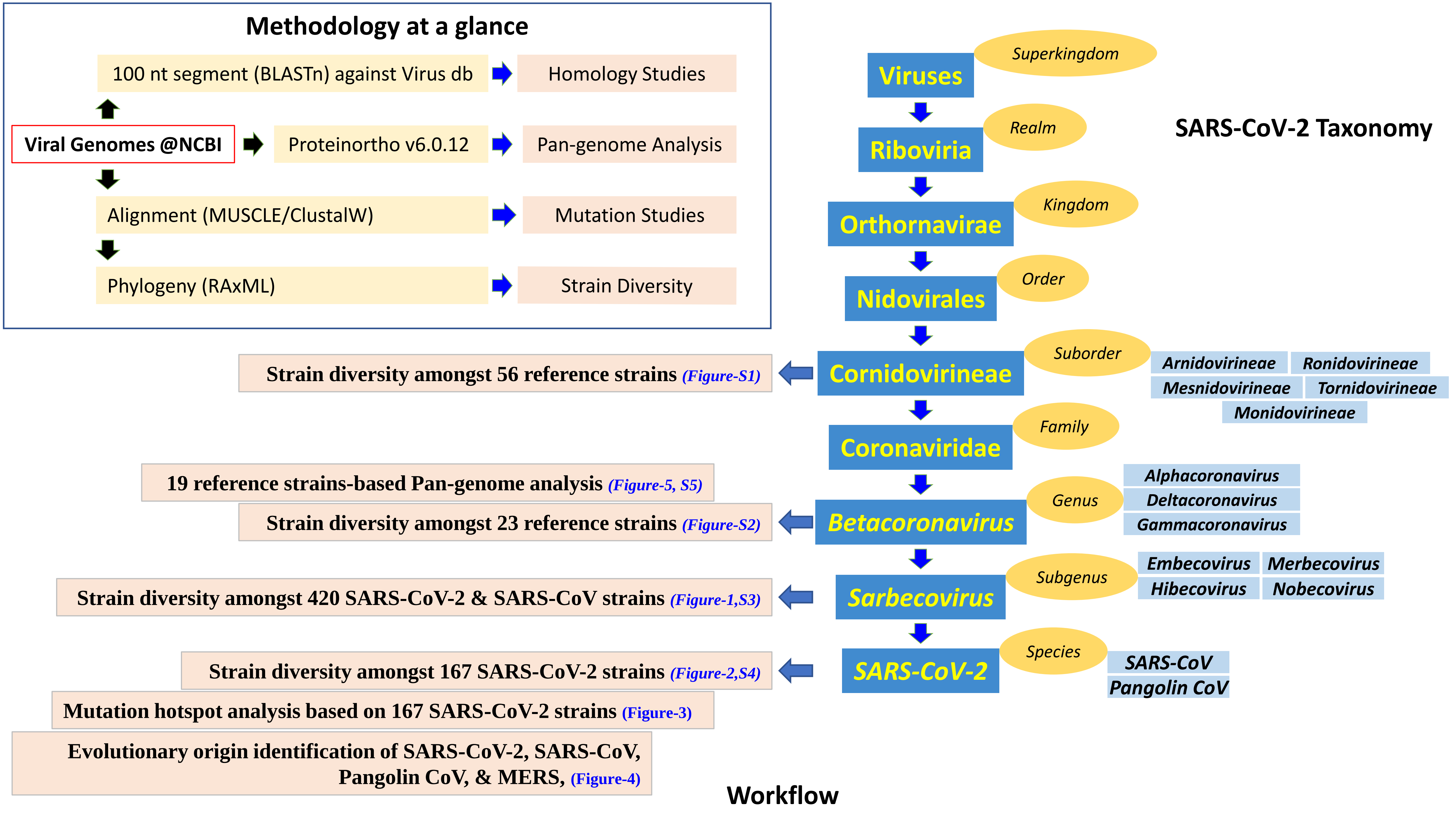
Methodology at a glance
100 nt segment (BLASTn) against Virus db
Homology Studies
Viral Genomes @NCBI
Proteinortho v6.0.12
Pan-genome Analysis
Alignment (MUSCLE/ClustalW)
Mutation Studies
Phylogeny (RAxML)
Strain Diversity
SARS-CoV-2 Taxonomy
Viruses
Superkingdom
Riboviria
Realm
Orthornavirae
Kingdom
Nidovirales
Order
Cornidovirineae
Suborder
Arnidovirineae
Ronidovirineae
Mesnidovirineae
Tornidovirineae
Monidovirineae
Coronaviridae
Family
Betacoronavirus
Genus
Alphacoronavirus
Deltacoronavirus
Gammacoronavirus
Sarbecovirus
Subgenus
Embecovirus
Merbecovirus
Hibecovirus
Nobecovirus
SARS-CoV-2
Species
SARS-CoV
Pangolin CoV
Strain diversity amongst 56 reference strains (Figure-S1)
19 reference strains-based Pan-genome analysis (Figure-5, S5)
Strain diversity amongst 23 reference strains (Figure-S2)
Strain diversity amongst 420 SARS-CoV-2 & SARS-CoV strains (Figure-1,S3)
Strain diversity amongst 167 SARS-CoV-2 strains (Figure-2,S4)
Mutation hotspot analysis based on 167 SARS-CoV-2 strains (Figure-3)
Evolutionary origin identification of SARS-CoV-2, SARS-CoV, Pangolin CoV, & MERS, (Figure-4)
Workflow
